# Ecological Specialization of Two Photobiont-Specific Maritime Cyanolichen Species of the Genus *Lichina*

**DOI:** 10.1371/journal.pone.0132718

**Published:** 2015-07-16

**Authors:** Rüdiger Ortiz-Álvarez, Asunción de los Ríos, Fernando Fernández-Mendoza, Antonio Torralba-Burrial, Sergio Pérez-Ortega

**Affiliations:** 1 Integrative Freshwater Ecology Group, Center of Advanced Studies of Blanes, Spanish Council for Research (CEAB-CSIC), Blanes, Girona, Spain; 2 Department of Biogeochemistry and Microbial Ecology, Museo Nacional de Ciencias Naturales (MNCN-CSIC), Madrid, Spain; 3 Institute of Botany, Karl-Franzens-Universität Graz, A-8010, Graz, Austria; 4 Biosfera Consultoría Medioambiental, Candamo, 5 Bajos, 33012, Oviedo, Spain; Field Museum of Natural History, UNITED STATES

## Abstract

All fungi in the class Lichinomycetes are lichen-forming and exclusively associate with cyanobacteria. Two closely related maritime species of the genus *Lichina* (*L*. *confinis* and *L*. *pygmaea*) show similar distribution ranges in the Northeast Atlantic, commonly co-occurring at the same rocky shores but occupying different littoral zones. By means of 16S rRNA and phycocyanin operon markers we studied a) the phylogenetic relationships of cyanobionts associated with these species, b) the match of divergence times between both symbionts, and c) whether *Lichina* species differ in photobiont association and in how geography and ecology affect selectivity. The cyanobionts studied are closely related to both marine and freshwater strains of the genus *Rivularia*. We found evidence of a high specificity to particular cyanobiont lineages in both species: *Lichina pygmaea* and *L*. *confinis* incorporate specific lineages of *Rivularia* that do not overlap at the haplotype nor the OTU levels. Dating divergences of the fungal and cyanobacterial partners revealed an asynchronous origin of both lineages. Within each fungal species, selectivity varied across the studied area, influenced by environmental conditions (both atmospheric and marine), although patterns were highly correlated between both lichen taxa. Ecological speciation due to the differential association of photobionts to each littoral zone is suspected to have occurred in marine *Lichina*.

## Introduction

Cyanobacteria are key components of ecosystems across Earth since the great oxygenation event (2.45–2.32 billion years ago) [1]. In addition to playing an important role in basic ecosystem processes, such as nutrient cycling and nitrogen fixation [2], they have also contributed to the development and diversification of eukaryotic life. Not only as the origin of the chloroplasts of all photosynthetic Eukaryota, but forming more open symbiotic systems with other organisms, from plants (gymnosperms, angiosperms, pteridophytes and bryophytes), protists (diatoms and dinoflagellates), animals (sponges, corals and ascidians) to fungi [3].

The symbiotic relationship established with lichen-forming fungi is the most relevant cyanobacterial symbiosis from evolutionary and environmental perspectives, accounting for *ca*. 10% of the total known lichen symbioses [4]. At least twenty cyanobacterial genera have been found to participate in lichen symbioses, including the most abundant representatives from the orders Chroococcales, Pleurocapsales, Stigonematales and especially Nostocales [5], with *Nostoc* being the most common and well-studied genus [6–8]. However, knowledge of cyanobacteria involved in many lichen symbioses is still poor and many strains and species remain to be discovered and properly analyzed [7,9].

Relationships between lichen-forming fungi and their photobionts are often categorized on the basis of their selectivity and specificity (see [10–13] for widely used definitions). Both high [9,14,15] and low [15,16] specificity toward their cyanobionts have been reported in lichen-forming fungi. On the other hand, high selectivity patterns [14,17,18] seem to co-occur with low selectivity strategies [19]. The high selectivity shown by many members of Peltigerales (Ascomycota) and the presence of certain cyanobiont strains shared by several mycobiont species has been interpreted as specificity at a community scale proposing the existence of lichen guilds [20]. This evolutionary structure has also been suggested for the tropical lichen-exclusive genus *Rhizonema* [9] a cyanobacterium not yet found in free living form. These contrasting patterns and the degree of specificity and selectivity in different lichens seem to vary across geographic scales [6,15,18,21,22], and in some cases may be better explained by environmental factors [23]. Overall, it seems that whether mycobionts and photobionts are generalists or specialists depend on geography, ecology, and their evolutionary histories. However, host specialization appears to be low at least at large geographic scales, diminishing the opportunities for coevolution out of the geographic mosaic hypothesis [22,24].

Most of the studied relationships between lichen-forming fungi and their associated cyanobacteria are those of species from Lecanoromycetes, the most diverse and abundant group of lichenized fungi. However, three other high-rank taxa are known to form stable symbioses with cyanobacteria: the classes Lichinomycetes and Eurotiomycetes [25] in the Ascomycota, and the order Agaricales [26,27] in the Basidiomycota. Interestingly, all the species in the class Lichinomycetes form symbioses with cyanobacteria. Despite some studies on the diversity of cyanobacteria associated with this class [28–31], so far no molecular studies regarding cyanobiont diversity and mycobiont specificity and selectivity are available in this group of lichenized fungi.

Species of Lichinales generally appear linked to harsh environmental conditions [32,33] such as deserts or rocky seashores, both physiologically stressful environments for an organism [34,35]. Two species from the genus *Lichina* C. Agardh, *L*. *confinis* (O.F. Müll.) C. Agardh and *L*. *pygmaea* (Lightf.) C. Agardh, usually co-occur in rocky seashores from temperate to cold areas in the North Atlantic Ocean, being especially abundant on the European coasts. However, *L*. *confinis* is able to reach higher latitudes with occurrences in Norway and Iceland, while the distribution of *L*. *pygmaea* reaches lower latitudes with records in the Canary Islands [36]. *Lichina confinis* and *L*. *pygmaea* are saxicolous fruticose lichens [36], although *L*. *pygmaea* is larger, shows flattened branches (cylindrical in *L*. *confinis*) and possesses a well-defined cortex structure that is not present in *L*. *confinis* [37]. These anatomical differences seem to be linked to their ecological niche. *Lichina confinis* is restricted to the supratidal zone, where it spends most of the time dehydrated or affected by sea spray. On the other hand, *L*. *pygmaea* occurs in the intertidal zone, where tidal forces, cycles of dehydration and temperature, and interspecific competition are more pronounced. Thus, in spite of the short distance separating both species, the niche is different due to the marked ecological gradient displayed in the seashores [38–40].

Pre-molecular studies about the identity of the photobionts associated with the lichen-forming genus *Lichina* resulted in mixed opinions. While some of the first lichen treatises from the early 1900’s identify them as members of the family Rivulariaceae [41,42], more recent works contend that *Lichina* photobionts belong to the genus *Calothrix* (Rivulariaceae) [4,43,44]. However, a molecular characterization of *Lichina* photobionts has not yet been conducted.

Since these two *Lichina* species from the seashore share similar broad-scale distribution ranges, though markedly different local-scale ecological niches, they may serve as a model to study the role of ecology and geography in photobiont selectivity in the Lichinomycetes. Furthermore, this study adds to our general understanding of cyano- and mycobiont relationships [7].

With this study we aim to: (1) clarify the taxonomic and phylogenetic placement of the cyanobacterial partners of coastal *Lichina* lichens, discussing the (2) possible temporal synchrony in the evolution of mycobionts and cyanobionts; (3) describe the degree of specificity and selectivity in photobiont use of each species, as well as their overlap; and finally (4) test the extent to which niche differentiation, local adaptation and symbiont partitioning contribute to the observed patterns of photobiont use, as well as the distribution of photobiont lineages along geographic and environmental gradients.

## Materials and Methods

### Sampling localities and collected taxa

Samples of *Lichina pygmaea* and *L*. *confinis* were collected in 32 localities in the Atlantic coasts of Europe and the Canary Islands (S1 Table). Specific permissions were not required, since *Lichina pygmaea* and *L*. *confinis* are not listed as endangered nor protected species in any official document. Sampling localities span in latitude between 28°N and 57°N, although a greater collecting effort was made to cover the southernmost portion of their distributional ranges, especially in the Atlantic and Cantabric coasts of the Iberian Peninsula.

Between 7 and 15 thalli were collected per locality. Samples were air dried until analysis, and later stored at the herbarium of the Real Jardín Botánico de Madrid (MA).

The specimens of *L*. *pygmaea* collected in the Canary Islands are not congruent with the other specimens both morphologically and genetically (fungal ITS, data not shown), suggesting the presence of an undescribed taxon in the region which we will tentatively address here as *Lichina ‘canariensis’* pending a complete taxonomic treatment of the taxon to be further developed in a later article.

In addition, we include in the study two free-living colonies of *Rivularia* collected in the Canary Islands, growing in the same coastal belt as *Lichina*, to discuss their relationship with the lichenized specimens.

### DNA extraction

For DNA extraction and sequencing we selected at least 3 specimens of each species per locality. The specimens were fragmented under the dissecting microscope excising small (*ca*. 4 mm^2^) pieces of thallus with the help of a sterile razor blade and a needle. These were repetitively cleaned and microscopically examined to eliminate any conspicuous trace of epiphytic organisms.

Samples were placed in 1.5 ml microcentrifuge tubes and DNA was extracted using either DNAeasy Plant Mini Kit (Qiagen) following manufacturer’s instructions or a modified version of the CTAB method [45]. DNA from extremely reduced thallus fragments was extracted using QIAamp DNA Investigator Kit (Qiagen).

### 16S rRNA and Phycocyanin operon amplification

Two different regions from the cyanobacterial genome were amplified. The V2-V6 regions of the 16S ribosomal RNA gene for cyanobiont identification and clade age estimation were amplified using the primers pair 106F [46] and 373R [47], and selected after removal of adjacent fragments of V1 region and ITS region. Reaction mix [47] and PCR conditions [46] were combined from the two original protocols of the primers.

The phycocyanin (PC) operon, including the intervening intergenic spacer (cpcBA-IGS, hereafter abbreviated as PC-IGS), was also amplified in order to study the genetic diversity along the latitudinal gradient as it has shown enough variability to distinguish between closely related samples [48–50]. The primer pair PCβF/PCαR [51] was primarily used following the conditions proposed by the authors. As amplification was unevenly successful, we adopted the primer pair RivR/RivF [50] with similar uneven success. Finally we designed a new primer pair using the software Primer-Blast [52]. The new specific primers PhyRivF (GCTATGTTACCTACGCKATG) and PhyRivR (TTGGACTTACCGCGAGAATC) together with PuReTaq Ready-To-Go PCR Beads (From GE Healthcare) produced successful amplification. Reactions were carried out with an initial step of 95°C, followed by 35 cycles (95°C for 1 min, 54°C for 30 seconds, and 72°C for 30 seconds) with a final step of 72°C for 15 min.

PCR products were purified using the UltraClean PCR Clean-up Kit (MOBIO Laboratories Inc). Both DNA strands were sequenced using the same primer pairs used in the amplification step. For the 16S region we used intermediate primers 781R [46] and 359F [47]. Samples were sequenced by Macrogen Inc. Laboratories (South Korea) and stored in Genbank under the accessions KR150498-KR150514 (16S rRNA) and KR606072-KR606140 (Phycocyanin sequences).

### Phylogenetic Analyses

Complementary sequence fragments were checked and collapsed into contigs using SeqMan software (Lasergene v 7.00, DNASTAR). Contigs were later aligned using ClustalW as implemented in BioEdit [53]. Alignments were further manually refined and collapsed to haplotypes using the online tool Fabox [54].

The adequacy of alternative substitution models was tested using jModeltest v2.1.4 [55]. The GTR+G substitution model with estimated base frequencies was estimated to be the optimum for both 16S and PC-IGS alignments based on AIC ranks.

For the simultaneous inference of the phylogenetic position of *Lichina* photobionts and dating the age of the clade we used a 16S rRNA alignment (1071 pb) including 17 sequences corresponding to the cyanobacterial clade E1 (as defined by [1], which contains mainly cyanobacterial divisions IV and V according to [56]), 51 sequences from the order *Nostocales* mostly corresponding to *Calothrix* and *Rivularia* taxa, and the 32 newly sequences obtained in this study (S2 Table). The new 32 sequences correspond to selected specimens representing each of the phycocyanin OTUs (delimited by means of bGMYC algorithm, see below). The phylogenetic tree was secondarily calibrated using a previously estimated age for clade E1 of 1.72 billion years ago (ya), with a 95% high probability distribution ranging from 1.28 to 2.25 billion ya [1] as a previous uniform prior constraint with a lognormal relaxed clock. Tree topology was simultaneously estimated using the Bayesian Markov Chain Monte Carlo method implemented in BEAST v1.7.5 [57] through the online platform CIPRES [58]. Two analyses were run independently for 50 million generations starting from a random tree, sampling trees every 2000 generations and deleting the first 10% of data as burn-in. We combined the runs using LogCombiner v1.7.5 (http://beast.bio.ed.ac.uk/LogCombiner) and determined stabilization of posterior distributions using TRACER v1.5. (http://tree.bio.ed.ac.uk/software/tracer/). Maximum likelihood (ML) analysis was implemented in RAxML v8.1.11 (Randomized Accelerated Maximum Likelihood) [59] through the RAxML blackbox on the CIPRES portal. ML searches were implemented using the GTRGAMMA substitution model. Bootstrap support was calculated based on 1000 replications. Those nodes with a bootstrap value (b) ≥70 were considered supported.

We estimated the divergence time of *L*. *confinis* and *L*. *pygmaea* fungi through a secondary calibration using a recently published time-calibrated phylogeny [60] with the estimated date of origin of Lichinomycetes as a previous uniform constraint (267 Million years ago (Mya); with 95% HPD intervals ranging between 204 Mya and 328 Mya ago) in the latest Lichinomycetes phylogeny [61] based on 18S. More recent work using a different set of fossils in Ascomycota [62] agreed with a late Permian origin of the Lichinomycetes lineage (274 (197–379) Mya). We used the same procedure and software as for the photobiont calibration.

Furthermore we used statistical parsimony [63] to generate an haplotype network from the PC-IGS region using the software TCS v.1.21 [64] reading gaps as a fifth informative character.

### Operational Taxonomic Unit (OTU) clustering

Clustering of photobiont OTUs on the PC-IGS haplotype dataset was carried out using two alternative implementations of the GMYC algorithm [65], which uses the differential distribution of intraspecific and interspecific branch lengths in ultrametric phylogenies to delimit evolutionary units. First we used GMYC as implemented in the package *splits*. We estimated alternative delimitations on an ultrametric maximum clade credibility tree based on single and multiple threshold models that we compared against a null model using likelihood ratio test [66,67]. Second, we used the recursive multitree approach implemented in the R package bGMYC [68] to incorporate phylogenetic uncertainty. The GMYC analysis was iteratively run on a subset of 1000 randomly chosen trees using a chain length of 50000 sampling steps, a burn-in of 40000 and a thinning parameter of 100. The results of all GMYC analyses are summarized in a matrix of pairwise co-assignment probabilities for each haplotype, shown as a heatmap. We developed a new method to obtain a consensus partition making use of k-medioids clustering [69] and optimum average silhouette width to estimate the optimum number of clusters. For this we used function *pamk* implemented in R package *fpc* [70] on the co-assignment matrix converted into its dissimilarity correlate.

### Haplotype diversity, genetic diversity and connectivity

Genetic diversity and geographic connectivity was calculated by pooling sampling localities into larger geographic regions: Canary Islands (CAN), Azores (AZO), Scottish and Welsh Atlantic coasts (UK), Brittany (FRA), Algarve (Alg), Galician West Coast (GAL), Bay of Biscay coast (CC2, CC1). The coast of the Bay of Biscay (longitude 1W to 8W) is split into two populations, CC1 and CC2. CC1 comprises all eastern localities, (1°W–5°W) which have a carbonate rich lithology while CC2 comprises all western localities (5°W–8°W) with predominantly acidic lithologies. The Galician localities (GAL) were treated separately from those of CC2, following their different biogeographic affinities [71].

Genetic connectivity between regions was evaluated using the distribution of PC-IGS haplotypes and OTUs and drawn into a map. The haplotypes shared between regions and species are summarized using a network produced in R and *Gephi* [72].

Genetic diversity measurements were calculated in DnaSP v.5 [73] for each geographic area and evaluated only if > 9 sequences were available. We measured the number of polymorphic sites (S, the number of positions in the sequence having more than one nucleotide per position), haplotype diversity (Hd, probability that two randomly chosen haplotypes are different within the sample) [74] and the nucleotide diversity (π, probability that two randomly taken nucleotides from the same position are different) [75].

### Environmental variables

Environmental variables were extracted using DIVAGIS V7.5 from WorldClim [76] and Bio-ORACLE [77], adding geographic location (latitude and longitude) and a categorical variable corresponding to substrate type (acid, calcareous and volcanic) (used variables listed in S3 Table). Variables were normalised (subtracting the mean of each data and dividing by the standard deviation for that variable) and tested with Draftsman pairwise scatter plots that samples were roughly symmetrically distributed across the range of each variable [78,79].

### Cyanobiont composition per locality

Similarity matrices between localities were generated for haplotypes and the different OTUs types considered. OTUs/haplotypes from free-living *Rivularia* were not included in these analyses. Using photobionts of each lichen species separately, analysis of similarities (ANOSIM) was performed with *a priori* geographical groups (10,000 permutations were run in each analysis). The contributions of OTUs/haplotypes to similarities within a group and differences between groups were analysed with SIMPER. All community analyses were conducted in PRIMER v6.1.6 (Primer-E) [78].

### Indirect gradient analysis of OTU and haplotype datasets

The relationship between environmental variables and photobiont communities was analysed using the BVSTEP routine of Primer v6.1.6. Spearman's rank correlation coefficient was used to link environmental matrix (Euclidean distance) with photobiont matrices (Sorenson’s index), and 10,000 permutations were run (ending criteria rho>0.95, delta rho<0.001) [78,80]. An exclusion criterion was used to avoid strong correlation between environmental variables (exclusion of one of the variables of the model if Euclidean distance between a pair of variables > 0.80). The most repeated environmental variables in the ten top models in the simulations for photobionts of each lichen species were selected and the model compared with another 10,000 simulations (different from those used to generate the models).

## Results

### PC-IGS sequence datasets

The final dataset of the phycocyanin operon comprises 205 specimens, 91 of which were sequenced from *L*. *pygmaea*, 94 from *L*. *confinis*, 18 from *L*. *‘canariensis’*, and two from free-living cyanobacterial colonies. The reduced sample sizes of the French locality and two localities from the Bay of Biscay (two sequences each) are due to the impossibility to obtain many specimens free of epiphyte contamination. The final alignment is 582 bp long, with 167 variable sites of which 150 are parsimony informative. All sequences, when compared with a BLAST query against NCBI databases, lie within the genus *Rivularia* and consistently retrieve three accessions (EU009171, EU009169 and EU009170) as closest matches.

### 16S rRNA phylogenetic relationships

The 16S dataset assembled incorporates 32 new sequences from 20 of the 22 OTUs intended (OTUs 5 and 20 failed to amplify). The alignment comprises 17 distinct new 16S rRNA ribotypes and a length of 1089 bp.

**Fig 1:**
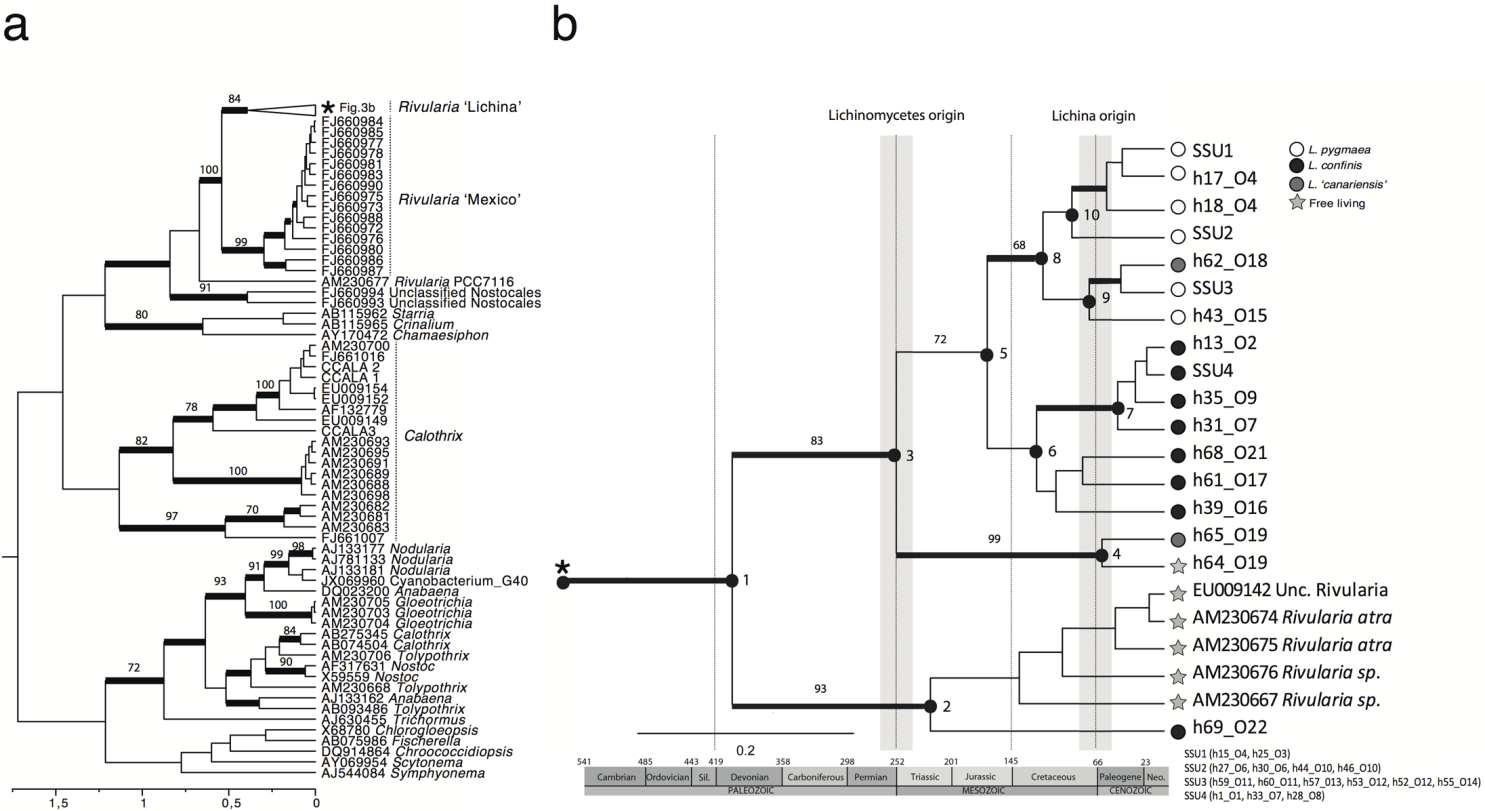
Dated maximum clade credibility tree for *Lichina* photobionts. Fig 1a shows the complete tree and Fig 1b depicts the *Rivularia* clade (both connected through an asterisk (*) symbol). The estimated divergence of Lichinomycetes and the genus *Lichina* is indicated in grey lines. The cyanobiont 16S rRNA unique haplotypes are named after the PC-IGS haplotype of the same sample, or SSU1–4 if collapsed from several phycocyanin haplotypes. Bold branches indicate pp ≥ 0.95. Maximum likelihood bootstrap values are indicated for relevant branches if value≈70 or higher. Estimated divergence dates for the complete tree are available in S1 Fig).

Cyanobiont ribotypes cluster together with members of the genus *Rivularia* (Fig 1A) in a well-supported clade (pp = 0.95, b = 100). A 16S sequence belonging to the genome of *Rivularia* PCC7116 is basal to two major supported groups of *Rivularia* sequences. The first clade comprises sequences from cultures obtained from Pozas Azules fresh water pond in Mexico [81]. The second clade contains all *Lichina* cyanobionts and free-living strains from Europe (Fig 1B). Within this clade two major groups are supported, one contains the ribotype h69_O22 from *L*. *confinis* (Wales, UK) which clustered together with *Rivularia* strains AM230674, AM230675, AM230676, and AM230667 from the Baltic Sea, in Finland [82] and EU009142 from the Alhárabe River, in Spain [50] with which it showed 98–99% similarity. The second group contains the remaining 16S ribotypes from *Lichina* cyanobionts and it may be divided in three subclades. The first shows the free-living cyanobacteria from Canary Islands (h64_O19) closely related to a lichen cyanobiont from the same region (h65_O19) (pp = 100, b = 99). The second subclade contains all *L*. *pygmaea* and one *L*. *‘canariensis’* (h62_O18) cyanobionts (pp = 0.98, b = 68). And the third subclade, with low statistical support, contains the majority of *L*. *confinis* cyanobionts. Thus, ribosomal DNA alone identifies a single split between the supratidal cyanobionts (*L*. *confinis*) and most intertidal cyanobionts (*L*. *pygmaea* and OTU 18 of *L*. *‘canariensis’*). Although this node shows low statistical support it has been always recovered, including neighbour-joining and maximum parsimony analyses (data not shown).

### Cyano- and mycobiont dated phylogenies

The calibrated phylogeny (Fig 1A and 1B, S1 Fig) shows a distinct pre-Paleozoic origin of lichen-associated *Rivularia* (569 (280–960) Mya). The split between two of the *L*. ‘*canariensis’* cyanobionts and the rest occurred at the end of the Permian period (250 (110–450) Mya). The likely break between intertidal and supratidal strains occurred during the mid Jurassic period with the supported clade of *L*. *pygmaea* photobionts (Node 5, 170 (70–290) Mya). Further, each of these groups diversified by the end of the Cretaceous and early Cenozoic (Nodes 6, 7, 8, 9 and 10 in Fig 1B). Ribotypes from the Canary Islands do not group together. On the other hand, the divergence time of *L*. *pygmaea* and *L*. *confinis* was estimated in 9,8 (5–25) Mya from a lineage that originates 62 (27–107) Mya ago (S2 Fig). Despite the high dispersion of the HPD intervals we can conclude that divergence of *Rivularia* at Node 5 (Fig 1B) occurred c. 100 million years prior to mycobiont divergence.

### Phycocyanin operon haplotypes

The collapse of the alignment resulted in 69 unique haplotypes, of which 38 are singletons and two are overrepresented: 1 (18 specimens of *L*. *confinis*) and 14 (16 specimens of *L*. *pygmaea*). Despite of the variation in geographic distribution of each haplotype, all haplotypes are exclusive and none is shared between the three *Lichina* species considered: *L*. *confinis*, *L*. *pygmaea* and *L*. *‘canariensis’*.

**Fig 2:**
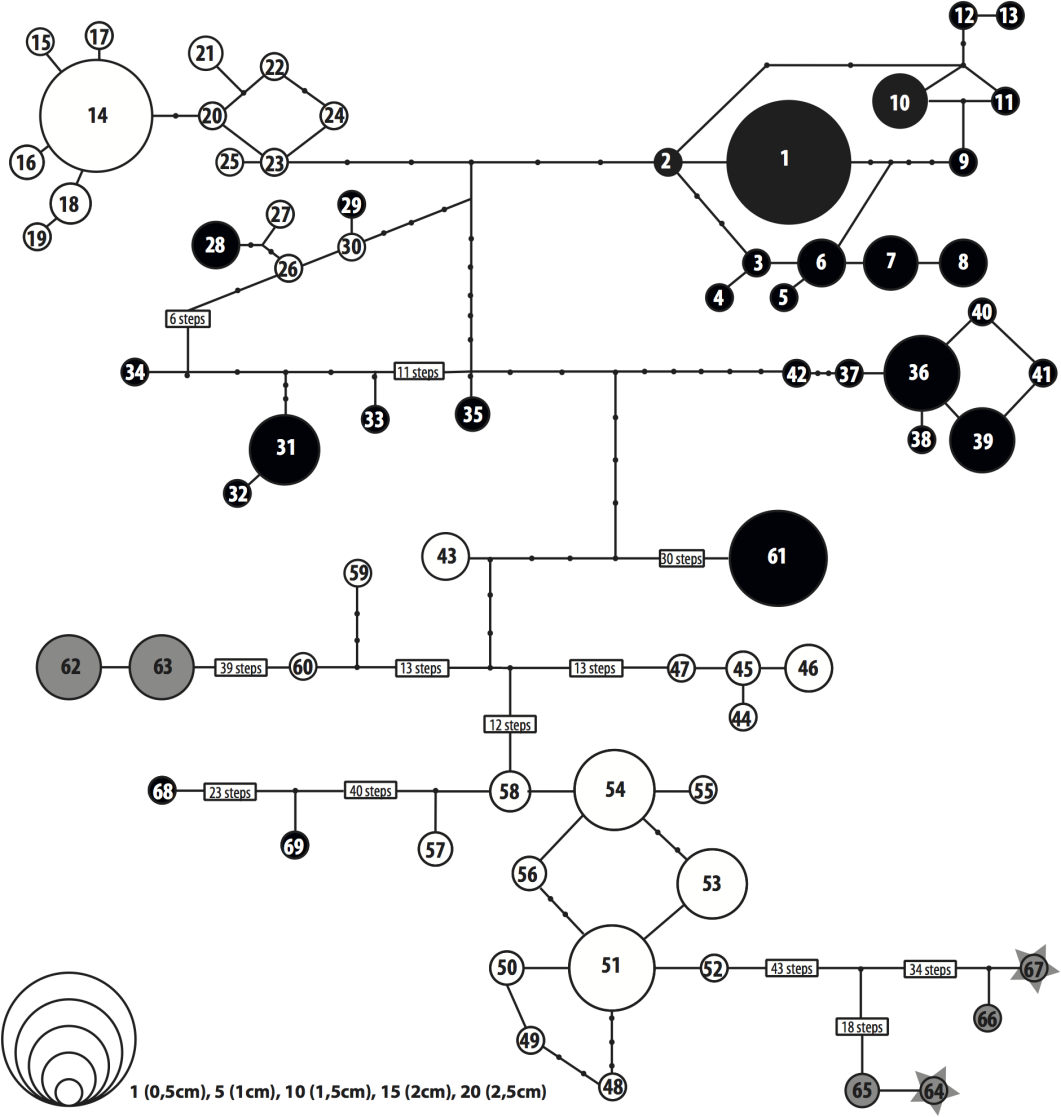
The 95% probability statistical parsimony network of *Lichina* cyanobionts based on PC-IGS sequences. Colors depict the mycobiont species (white for *L*. *pygmaea*, black for *L*. *confinis*, grey for *L*. *‘canariensis’*, and starred-grey for free-living *Rivularia*) and size of the circles the number of sequences that each haplotype contains. Large numbers of connection steps between haplotypes are indicated in boxes to improve conceptualization

**Fig 3:**
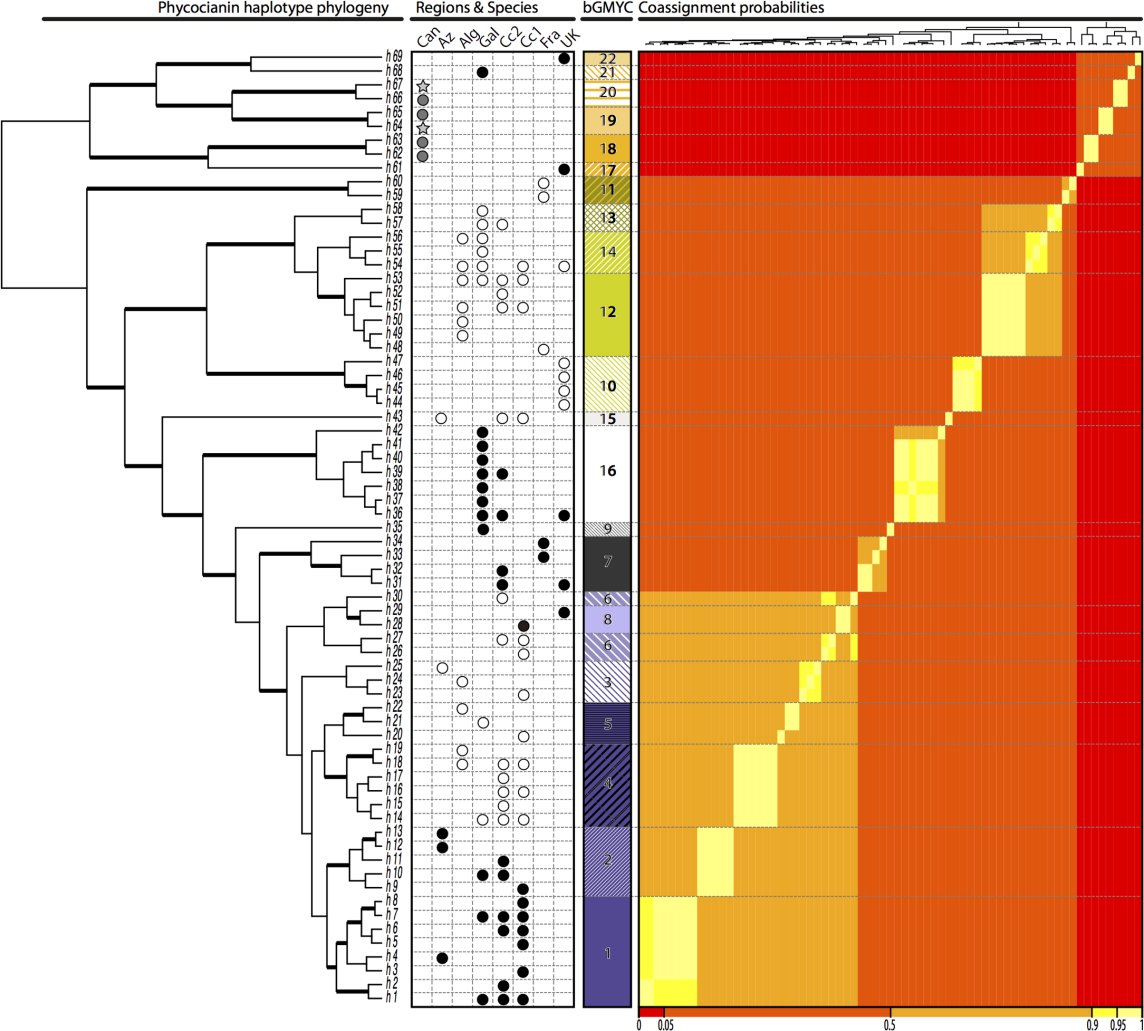
Maximum clade credibility tree based on Bayesian inference of the phycocyanin operon. Geographic origin and fungal partner of each sample is indicated in a dot-matrix (*L*. *confinis-*white-, *L*. *pygmaea-*black*-*, *L*. ‘*canariensis*’*-*grey- or free-living *Rivularia–*starred-grey-). Median partitions from the bGMYC model for OTU-clustering are indicated (OTUs 1 to 22) together with a heatmap-like graph that represents the co-assignment probability matrix of OTU assignment (colors ranging from red for the lower probabilities and intense yellow for the higher probabilities).

The haplotypes found in the different species of *Lichina* do not form coherent clades in the phycocyanin reconstructions (Figs 2 and 3) as they do in the 16S phylogeny. However, the *Rivularia* haplotypes found in each *Lichina* tend to be aggregated in sub-networks exclusive to each species (Fig 2), except the closely related haplotypes 26, 27 and 30 which were found in *L*. *pygmaea*, while 28 and 29 were found in *L*. *confinis*.

The parsimony network (Fig 2) is formed by a central sub-network, which includes the majority of specimens, and five peripheral ones separated by 35–58 steps from the closest haplotype and distant from each other. This topology is coherent with the six well-supported clades separated by long branches in the Bayesian phylogenetic tree (Fig 3). Three of these peripheral clades comprise the free living and lichenized samples collected in the Canary Islands (*L*. *‘canariensis’*). The other two peripheral clades include haplotypes sequenced from *L*. *confinis*: one includes haplotype 61 (OTU 17, Figs 2 and 3) exclusive to Scotland and the other the distantly related haplotypes 68 (OTU 21) from Galicia and 69 (OTU 22) from Wales.

### Descriptive metrics of genetic diversity

Descriptive metrics of genetic diversity and variability in the cyanobionts of the three *Lichina* species and across geographic regions are summarized in Table 1. The three species considered in the study show similar overall levels of nucleotide (π) and haplotype diversities (Hd) (Table 1), although haplotype diversity in *L*. *‘canariensis’* is significantly lower as a result of the smaller sample size (Table 1).

**Table 1:** Summary of genetic diversity measures for the different geographic areas and *Lichina* species (Lp: *Lichina pygmaea*; *Lc*: *L*. *confinis*; Lcan: *L*. *‘canariensis’*). For each area/species we display the number of sequences (Nseq), the number of haplotypes (Nhap), the number of polymorphic sites, haplotidic diversity (Hd) and nucleotidic diversity (π).

	****Lp/Lc/Lcan****	****CC1****	****CC2****	****Gal****	****Azo****	****UK****	****FR****	****Alg****	****Can**** ^1^
**Nseq**	91/94/18	26/27	13/22	19/22	3/3	9/18	3/2	18/-	20
**Nhap**	31/29/5	12/9	11/9	8/11	2/3	4/6	3/2	9/-	6
**S**	74/108/98	45/15	45/33	38/82	20/10	26/96	31/5	42/-	102
**Hd**	0.93±0.01/0.93±0.013/0.72±0.07	0.76±0.08/0.80±0.06	0.96±0.05/0.9±0.03	0.88±0.04/0.90±0.04	0.67±0.31/1±0.27	0.75±0.11/0.72±0.09	1±0.27/1±0.5	0.80±0.09/-	0.77±0.07
**π**	0.040/0.03/0.040	0.027/0.008	0.0350/0.025	0.025/0.031	0.024/0.011	0.011/0.054	0.037/0.01	0.021/-	0.056

^1^ includes two sequences from free-living organisms belonging to the Canary Islands; therefore these numbers refer to the Canary Islands strains as a whole.

The geographic distribution of cyanobiont diversity per species within each of the eight geographic areas studied is quite homogeneous, despite differences in sample sizes. It is remarkable that the highest Hd was found for both species in CC2 (0.96 and 0.9) and the lowest in UK (0.79 and 0.74). The highest π for *L*. *confinis* is observed also in CC2 (0.035), but for *L*. *pygmaea* it is observed in UK (0.054). The lowest π for L. *pygmaea* is found in UK (0.011) and for *L*. *confinis* in CC1 (0.008).

### Delimitation of OTUs based on the PC-IGS

**Fig 4:**
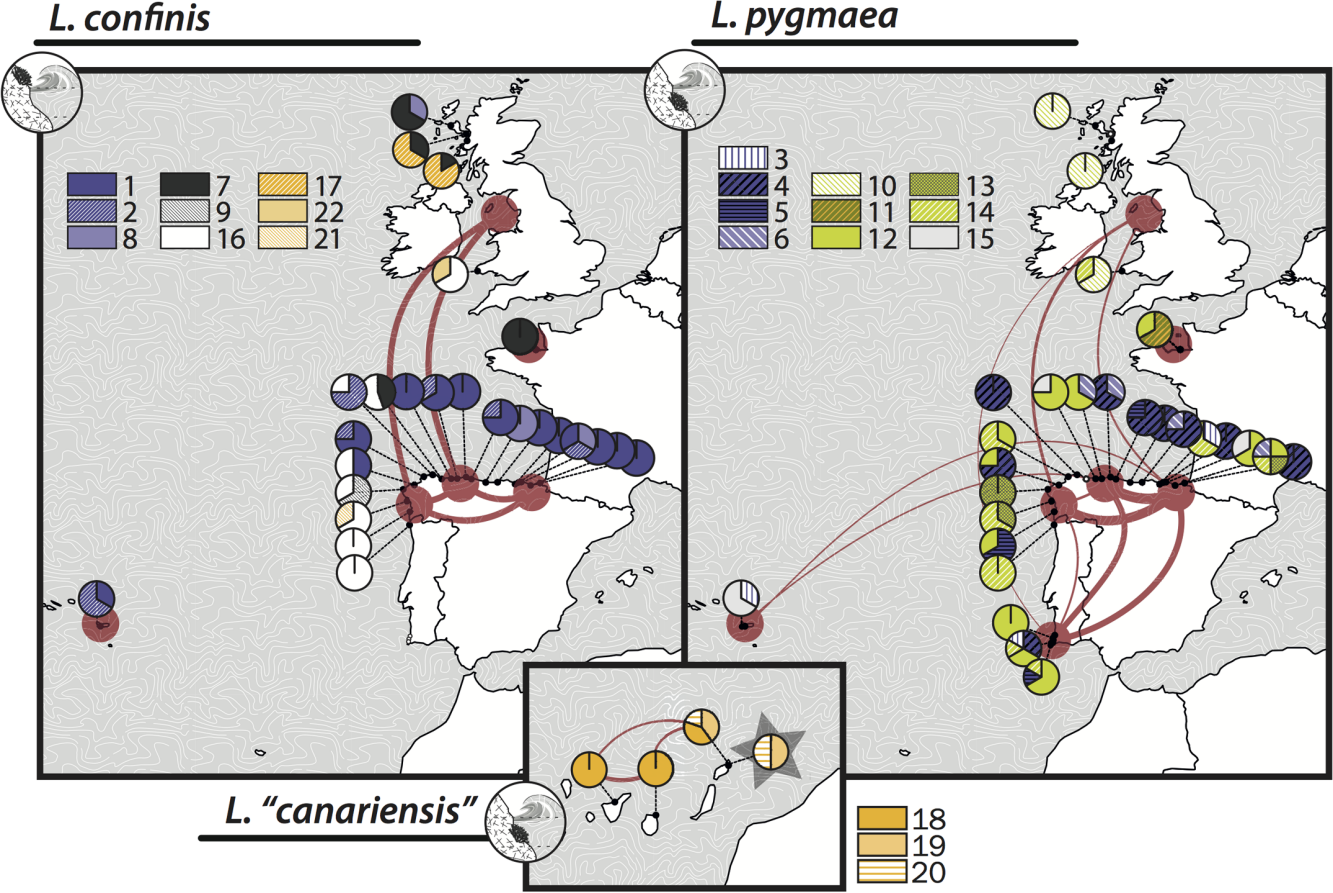
Map of geographic distribution of phycocyanin OTUs and haplotypes. The pie charts show the proportion of each OTU per sampling locality. Color codes aim to reflect the presence of the main PC-IGS clades and are coherent with those of Fig 3. Haplotype connectivity between geographic regions is shown using a network. The width of the edges is proportional to the number of shared haplotypes, ranging from one to seven. The figure was generated with rworldmaps based on the public database Natural Earth (http://www.naturalearthdata.com)

The GMYC single and multiple threshold analyses were statistically different from the null model and retrieved 14 (confidence interval = 12–20) and 20 (confidence interval = 15–25) OTUs respectively (Table 2). While most OTUs were coherent with the clade structure found on the haplotype network, others were strongly biased by enforcing a single topology with some ill-supported clades (data now shown). We found that the bGMYC multitree implementation accommodated our dataset better by allowing us to incorporate phylogenetic uncertainty as well as uncertainty in the GMYC itself. The resulting co-assignment matrix is shown as a heatmap in Fig 3. Finally the use of k-medioids clustering and an optimum average silhouette width criteria allowed us to provide an analytical consensus partition without having to impose arbitrary threshold values on the co-assignment matrix as previously suggested [68]. The consensus partition infers 22 OTUs, which were used as units for downstream ecological analyses. The relation between haplotypes and OTUs, their geographic and intraspecific distribution is shown in Fig 3 and in Fig 4.

**Table 2:** Summary of the single and multiple thresholds GMYC analyses showing the number of groups retrieved, the confidence intervals, the values of likelihood of the null models, and the their likelihood ratio tests.

Method	OTU clusters	Confidence intervals	Null model Likelihood	GMYC model Likelihood	Likelihood ratio
Simple	14	12–20	487.751	497.2483	18.99447***
Multiple	20	15–25	487.751	499.4952	23.48842***

*** indicates the ratio test is statistically significant.

### Geographic structure of cyanobiont association

The three taxa of *Lichina* studied associate with non-overlapping cyanobiont pools. Each species incorporates a different set of haplotypes and OTUs. Even in localities where *L*. *confinis* and *L*. *pygmaea* associate with closely related (PC-IGS) cyanobionts, the cyanobionts of the intertidal ‘*Lichina canariensis’* are very different from those associated with the other two species. Despite its isolation from the remaining region, the Canary Islands show interregional connectivity between the three islands studied (Tenerife, Gran Canaria and Lanzarote) at haplotype and OTU levels (Figs 2 and 4). The OTU 18 is prevalent in all Canarian localities but was not found free-living. In addition, OTUs 19 and 20 incorporate haplotypes from lichenized and free living *Rivularia* collected in Lanzarote. The geographic structure of cyanobiont association in *L*. *confinis* and *L*. *pygmaea* is highly distinct. In both taxa the British and French localities have different OTUs, and show a strong pattern of genetic connectivity between Iberian coasts, but the extent of this connectivity and degree of overlap between regional cyanobiont pools are quite different.

*Lichina confinis* shows a highly structured pattern of cyanobiont distribution. Haplotype sharing is geographically restricted. Seven haplotypes are shared between at least two regions, five of which are restricted to the Iberian coast (GAL, CC1 and CC2) and two shared between the westernmost Galician and Asturian coasts (GAL and CC1) and the British region. In terms of OTUs, there is a gradual eastwards replacement of OTUs along the coast of the Bay of Biscay. The Galician coast is dominated by OTU 16, which is replaced by OTUs 8 and the closely related OTUs 1 and 2. These two are also found in Azores. In addition to the presence of the widespread OTU 1 in Scotland, OTUs 17 and 22 are exclusive in the British coasts, while OTU 7 is also found in France. Similarity analyses on OTU composition allow us to quantify the observed differences in cyanobiont structure between regions (Tables 3 and 4). Differences between other regions and UK are mainly due to OTU 7 (23.13%), OTU 17 (15.90%,), and OTU 1 (20.31%, absent in UK, and present in 63% of the northern Spain sampling sites). OTU 1 is less frequent in Galicia (33% of the sampling sites) than in the rest of northern Spain (60% in CC2 and 88% in CC1). Differences between Galicia and the rest of northern Spain (Table 3) are mainly due to OTU 16, which is more frequent in Galicia and absent in CC1. Together with OTU1, both account for 66.22% of the differences between Galicia and CC1, and 58% of the differences between Galicia and CC2. Interestingly, while Galicia and CC1 differ significantly in cyanobiont structure, CC2 is not significantly different from either of them.

**Table 3:** Differences between geographic areas for *L*. *confinis* OTUs according to ANOSIM similarity analysis.

	*Lichina confinis*					
	****UK****	****France****	****CC1****	****CC2****	****Galicia****	****Azores****
**UK**		-0.375	0.699	0.313	0.490	0.500
**France**	n.s.		0.786	0.280	0.733	—
**CC1**	**	n.s.		0.091	0.497	-0.036
**CC2**	n.s.	n.s.	n.s.		0.119	-0.360
**Galicia**	*	n.s.	**	n.s.		0.367
**Azores**	n.s.	—	n.s.	n.s.	n.s.	

Upper matrix shows R statistic values, and lower matrix their significances (n.s. = non significant; * = p<0.05; ** = p<0.01). Global R Statistic = 0.366, **.

**Table 4:** Differences between geographic areas for *L*. *pygmaea* OTUs according to ANOSIM similarity analysis.

	*Lichina pygmaea*						
	****UK****	****France****	****CC1****	****CC2****	****Galicia****	****Algarve****	****Azores****
**UK**		**0.500**	**0.428**	**0.375**	**0.369**	**0.602**	**0.500**
**France**	**n.s.**		**0.286**	**0.060**	**0.067**	**0.222**	**—**
**CC1**	**	**n.s.**		**-0.003**	**0.141**	**0.071**	**0.268**
**CC2**	*	**n.s.**	**n.s.**		**0.163**	**0.046**	**0.120**
**Galicia**	*	**n.s.**	**n.s.**	**n.s.**		**-0.182**	**0.467**
**Algarve**	**n.s.**	**n.s.**	**n.s.**	**n.s.**	**n.s.**		**1**
**Azores**	**n.s.**	**—**	**n.s.**	**n.s.**	**n.s.**	**n.s.**	

Upper matrix shows R statistic values, and lower matrix their significances (n.s. = non significant; * = p<0.05; ** = p<0.01). Global R Statistic = 0.200, **.

For *L*. *pygmaea* the pattern of haplotype sharing is more complex, with nine haplotypes being shared among six of the eight regions studied (Fig 3). Haplotype connectivity is strong within the Iberian coasts, this time including the Algarve. The number of haplotypes shared between the Iberian regions and the rest ranges between one and two when haplotypes are shared. Compared to *L*. *confinis*, cyanobiont OTUs in *L*. *pygmea* are more widespread and more evenly distributed across regions, with the exception of the British coasts where the exclusive OTU 10 is dominant. The ANOSIM similarity analysis of cyanobiont distribution reinforces the pattern of differentiation between the northern Spanish coast (CC1+CC2+Galicia) and Britain (Table 4). The differences are mainly due to OTU 10 (29.17%), OTU 4 (20.69%, absent in the UK) and OTU 12 (12.85%, absent in UK). Other OTUs present in northern Spain and absent in the UK contributed at a lower scale to community structure.

Summing up, both species show an emerging pattern in which the specimens collected in the North-oriented coasts of the Bay of Biscay tend to use different cyanobiont lineages than the more West-facing Atlantic coasts of Galicia, Algarve, Wales and Scotland. Correlation analysis between locality similarity matrices showed a weak but statistically significant correlation between distributions of photobionts from *L*. *confinis* and *L*. *pygmaea* (R Spearman = 0.202, p < 0.001**). Finally, the apparent divergence between the French and Azores localities and the rest should be interpreted with care due to their smaller sample sizes.

### Effect of environmental variables on community structure

Using OTUs from *L*. *pygmaea* we generated a unique model (98.9% of the simulations) that used four environmental variables to explain biotic similarities among localities. These variables were temperature air range, sea surface maximum temperature (°C), substrate, and seawater phosphate. Correlation was relatively low (Rho = 0.366) but statistically significant (p<0.01). The results using haplotype composition were coherent, although the models incorporated temperature air range, substrate and sstmean (instead of maximum) (S1 File).

The best models explaining the OTU composition of *L*. *confinis* used between 4 and 6 variables, with correlations between 0.588 and 0.596 (S4 Table). The most frequent model (59.04% of the simulations) had a medium-high correlation (Rho = 0.595) and was statistically highly significant (p<0.001) using five environmental variables: sea surface temperature, cloud fraction (max), photosynthetically available radiation (medium), dissolved oxygen in seawater and water clarity. The first three appear in 80–100% of the generated models, accounting for the 74.8–100% of the simulations. Using haplotype variables related to temperature, cloud fraction, or clarity were also obtained, with similar correlation values (S1 File).

## Discussion

### Phylogenetic placement of *Lichina* cyanobionts

The genus *Lichina* associates specifically with cyanobacteria of the family Rivulariaceae [83]. Using a fragment of the 16S ribosomal RNA gene, we confirmed that the cyanobionts of the *Lichina* species studied are closely related to both marine and freshwater strains of the genus *Rivularia* [81,82]. Although *Calothrix* or *Dichothrix* have has been traditionally assumed to be the photobionts of *Lichina* species [4,36,44,84], *Rivularia* was proposed as a photobiont in 1874 [85]. Recent phylogenetic surveys of the Rivulariaceae [50,81,82] found that the classification of Rivulariacae [81] is complicated because of homoplasy in morphology-based delimitations and by evidence of the dependence of morphological characters on growth conditions (e.g. differential phosphorous supply [50]). Some authors had already noted the lack of resemblance between *Lichina* cyanobionts and free-living *Calothrix* [43], which tend to form individual filaments or ill-defined colonies.

### Incongruent phylogenetic signal between 16S rRNA and PC-IGS

The two loci used, 16S rRNA and PC-IGS, show incongruent topologies in the phylogenetic reconstructions (Figs 1, 2 and 3). In 16S (Fig 1) the majority of cyanobionts sequenced from *L*. *confinis* and *L pygmaea* group in well-supported, reciprocally monophyletic clades. However, the pattern in PC-IGS is more complex (Figs 2 and 3). The cyanobionts of *L*. *pygmaea* and *L*. *confinis* form separate clades but are not reciprocally monophyletic, and intergrade across the phylogeny. In both markers, the cyanobionts of *L*. *‘canariensis’* and the free-living specimens of *Rivularia* collected in the Canary Islands form a separate and divergent clade, except for sequence h62_O18, included in the *L*. *pygmaea* group in the 16S phylogeny.

Finding incongruent phylogenetic signals between loci was viewed as a problem in early phylogenetic studies; incongruence between loci is now commonly observed, as datasets grow wider and deeper, including growing numbers of specimens and markers. Incongruences appear from gene duplication, gene losses, horizontal gene transfers or incomplete lineage sorting [86,87]. Such incongruent signals are common among surveys of cyanobionts (e.g. 16S and trnL in [6,16,88,89]) and free-living cyanobacteria [90]. The ribosomal 16S rRNA gene has been successfully used to recover phylogenetic signal at large time scales but it is regarded as too uninformative to interpret recent events [18,20,91] at species and intraspecific levels, for which the use of other loci such as rbcL, tnrL or PC-IGS are more appropriate [7,50].

### Availability, specificity and selectivity in cyanobiont assembly

The observed patterns of photobiont association probably result from multiple processes [7,13], such as dispersal limitation, the availability of symbiotic partners, the specificity to certain groups or the selectivity of environmentally fit lineages. In turn, these processes might have implications at ecological and evolutionary timescales [13,92].

We observed that each species of *Lichina* incorporates a specific group of *Rivularia* cyanobionts, which do not overlap between species at any level of genetic complexity (haplotypes and OTUs). It is striking that cyanobiont haplotypes are shared between separated regions within *L*. *confinis* and *L*. *pygmaea* (network in Fig 4.), while specimens growing a few meters apart in the intertidal and supratidal zones consistently associate with different cyanobionts. Green algal lichens can also show high specificity to certain algal groups by potential local adaptation, as shown by recent research [93,94].

The divergence in photobiont association between closely related species occupying different coastal zones had never before been described in lichens. However, it is coherent with the observation that some lichens are highly selective [18,22] with their symbiotic counterparts and this may modulate their symbiont assembly in response to environmental factors [95,96] at ecological and evolutionary timescales [97]. Many alternative hypotheses can be formulated to explain the observed patterns of photobiont association. It is possible that *L*. *confinis* and *L*. *pygmaea*, both sexually reproducing species and hence establishing new associations each generation, draw symbionts from a shared coastal pool of available *Rivularia* species, and that each chooses certain symbionts in terms of i) genetic compatibility (specificity) or ii) ecological fitness (selectivity). However it is also possible that iii) differences in cyanobiont availability between both coastal zones might also contribute to the overall pattern and represent the most important factor shaping the observed pattern.

Given the markedly different ecologies of lichen species that live only a few meters apart, in an environment where vertical zonation is the main pattern of differential colonization for single species and littoral assemblages [98], we believe that the presence of two differentiated *Rivularia* pools originated through a process of ecological speciation [99] for each *Rivularia* lineage (Fig 1B). Ecological speciation has been repeatedly reported in the different zones of the coastal environment for macroorganisms like the sea snail *Littorina* [100,101], *Polychaeta* worms [102] or different types of littoral assemblages [98]. Furthermore, ecological speciation can happen in prokaryotes in an analogous way to macroorganisms [103], with functional capabilities being correlated with the microorganism’s habitat breadth [104]. In microorganisms, ecological speciation often appears linked to extreme conditions and results in daughter species or strains with restricted ecological ranges [105,106]. The two coastal zones where *Lichina* species and *Rivularia* lineages are found differ in many ecological aspects and support very different biotas. Further studies on other Lichinomycetes linked to harsh conditions like arid or semiarid areas [28] might also reveal a high level of specialization with their cyanobionts.

### Synchrony of mycobiont and cyanobiont evolution

Divergence time estimates for the split between *L*. *confinis* and *L*. *pygmaea* and between supratidal and intertidal *Rivularia* lineages do not coincide (Fig 1B, S1 Fig and S2 Fig), suggesting asynchrony between cyanobiont and mycobiont diversification events. The onset of the linage containing most of the *Rivularia* strains found in *Lichina* dated from the Paleozoic era, and seems to diversify into the intertidal and supratidal clades during the Mesozoic, a period characterized by Pangaea rifting and high-rate sea-level change [107]. However, the major radiation of *Rivularia* ribotypes is likely to have occurred by the end of the Cretaceous, when the second biggest mass extinction took place, matching the appearance of the *Lichina* lineage. Extreme climatic events seem to be related with the evolutionary history of the Lichinomycetes, as the onset of the lineage [60,62] has been estimated to occur close to the Permian-Triassic mass extinction, the largest known extinction that matches the ‘fungal spike’ and the origin of wide desert areas [108]. Thus, this asynchronous diversification between bionts suggests the existence of two ecologically differentiated cyanobiont pools, prior to the appearance of *Lichina*. Further, in spite of the large time windows recovered for each lineage, providing divergence times for the two partners of a symbiosis proves to be extremely useful to add evolutionary information of the symbiosis into myco-photobiont studies.

### Geographic structure of cyanobiont assembly

Within species we observed a pattern of cyanobiont selectivity across the area studied. The pattern differs only slightly between *L*. *confinis* and *L*. *pygmaea*, as indicated by the significant correlation between the regional similarity matrices (Tables 3 and 4, and Fig 4). In both cases there is a clear differentiation between the Northern Spanish coast and the Scottish and Welsh localities. This divergence can reflect differences in cyanobiont availability but also of preferential association with certain lineages in environmentally divergent areas.

In the case of *L*. *confinis*, there is a significant difference between Galicia and CC1, but both partially mix with CC2, suggesting an ecotone or a barrier that contains OTUs from both regions. A similar pattern has been seen for the estuarine seaweed *Fucus ceranoides* in the same region [71]. We found similar patterns using haplotypes rather than OTUs (S1 File).

In the intertidal *L*. *pygmaea*, genetic connectivity between regions is much stronger, and there is little differentiation between different coastal areas in the Iberian Peninsula.

Specimens from the two studied Macaronesian archipelagos showed quite different patterns. Although PC-IGS haplotypes recovered from Azores are endemic (except hap 43 from *L*. *pygmaea*, which is also found in the north of the Iberian Peninsula) they cluster within OTUs spread along the Iberian Peninsula coast. On the other hand, the Canary Islands showed not only endemic haplotypes and OTUs, but also are the most deviant haplotypes to the rest of sequences gathered in this study. The Canary Islands usually show a high number of endemic organisms due to their complex geological history as well as their physical and climatic heterogeneity [109–111]. The high genetic diversity found in the comparatively small sample size studied, supports the idea of oceanic islands as sources of biodiversity rather than the ‘end of colonization road’ [110,112]. This high contrast regarding genetic diversity and endemicity between the Canary Islands and Azores archipelagos likely reflects patterns already described for flowering plants and lichens where a high and low number of examples of inter-island allopatric speciation exist respectively [110,113].

### Correlation with environmental variables

The models generated for the *Rivularia* cyanobionts of *L*. *confinis* and *L*. *pygmaea* are coherent with the observations made on their geographical distributions. The models correlating environmental variables with the cyanobiont structure of both species are similar. Both species distributions are shaped by air temperature and sea temperatures, as previously observed for inland cyanobacteria [34,114], reinforcing the observation of a gradient of cyanobiont structure coherent with the influence of the Iberian Poleward Current in the North-western Iberian coasts. Also, both species are affected by nutrient content (phosphate for *L*. *pygmaea cyanobionts*, and water clarity as an indicator of nutrient content [115,116] for *L*. *confinis*). Further, it is surprising that the pH of the rock substrate arises as a factor only for *L*. *pygmaea* cyanobionts.

The supralitoral belt is a drier and sun-exposed environment. Therefore we expected to find a signal for humidity [40] and light-related variables [117]. All the models for *L*. *confinis* cyanobionts included dissolved oxygen content as an important explanatory variable, which may be an indicator of sea spray intensity cause by wave splash in choppy waters, and therefore higher saline water availability. Regarding light, models revealed photosynthetic available radiation (PAR) as an important factor, suggesting that certain OTUs might be adapted to different light levels.

Taken all together, it seems that the contribution of niche differentiation, local adaptation and symbiont partitioning contribute to the observed patterns of photobiont association, as well as the distribution of photobiont lineages along geographic and environmental gradients.

## Conclusions

This first molecular study of cyanobionts from a Lichinomycetes lineage shed light on the phylogenetic affiliations of the photobiont of marine *Lichina* species, which we assigned to the genus *Rivularia*. A high degree of ecological specialization, as cyanobiont haplotypes are linked to a single *Lichina* and never shared between species, points to the existence of two independent ecologically differentiated pools, in the supratidal and intertidal zones. Asynchrony in the divergence dates of *Lichina* and *Rivularia* OTUs suggest a lack of ancient coevolutionary history between the two symbionts. Low connectivity exists between the UK and the Northern Spanish coast, where we found signs of a potential ecotone. The Canary Islands showed a completely different community composition and a likely and still undescribed *Lichina* species. Finally, the distribution of *Rivularia* populations along the latitudinal gradient is highly associated with environmental variables. We encourage further studies in the Lichinomycetes group to extend the knowledge of cyanobiont distribution and specificity that we have started to unveil.

## Supporting Information

S1 Fig Time calibrated phylogeny based on cyanobacterial 16S rRNA. (PDF)

S2 Fig Time calibrated phylogeny of Lichinomycetes based on 18S rRNA and their sequence accession numbers. (PDF)

S1 File Effect of environmental variables on community structure (Haplotype level). (DOCX)

S1 Table Sampling locations of *L*. *confinis*, *L*. *pygmaea*, and *L*. *‘canariensis’* including the number of sequences used per locality and the substrate information. (XLSX)

S2 Table Accession numbers used in the 16S phylogeny. (TXT)

S3 Table Environmental variables used to generate the explanatory models of *L*. *confinis* and *L*. *pymaea* cyanobionts. (DOCX)

S4 Table Generated environmental models explaining the OTU distribution of *L*. *confinis* cyanobionts. (DOCX)
