# Supplementary figures and images for: Ecological Specialization of Two Photobiont-Specific Maritime Cyanolichen Species of the Genus Lichina
Source: PLoS One. 2015 Jul 16;10(7):e0132718. doi: 10.1371/journal.pone.0132718 (PMC4504470; doi:10.1371/journal.pone.0132718)

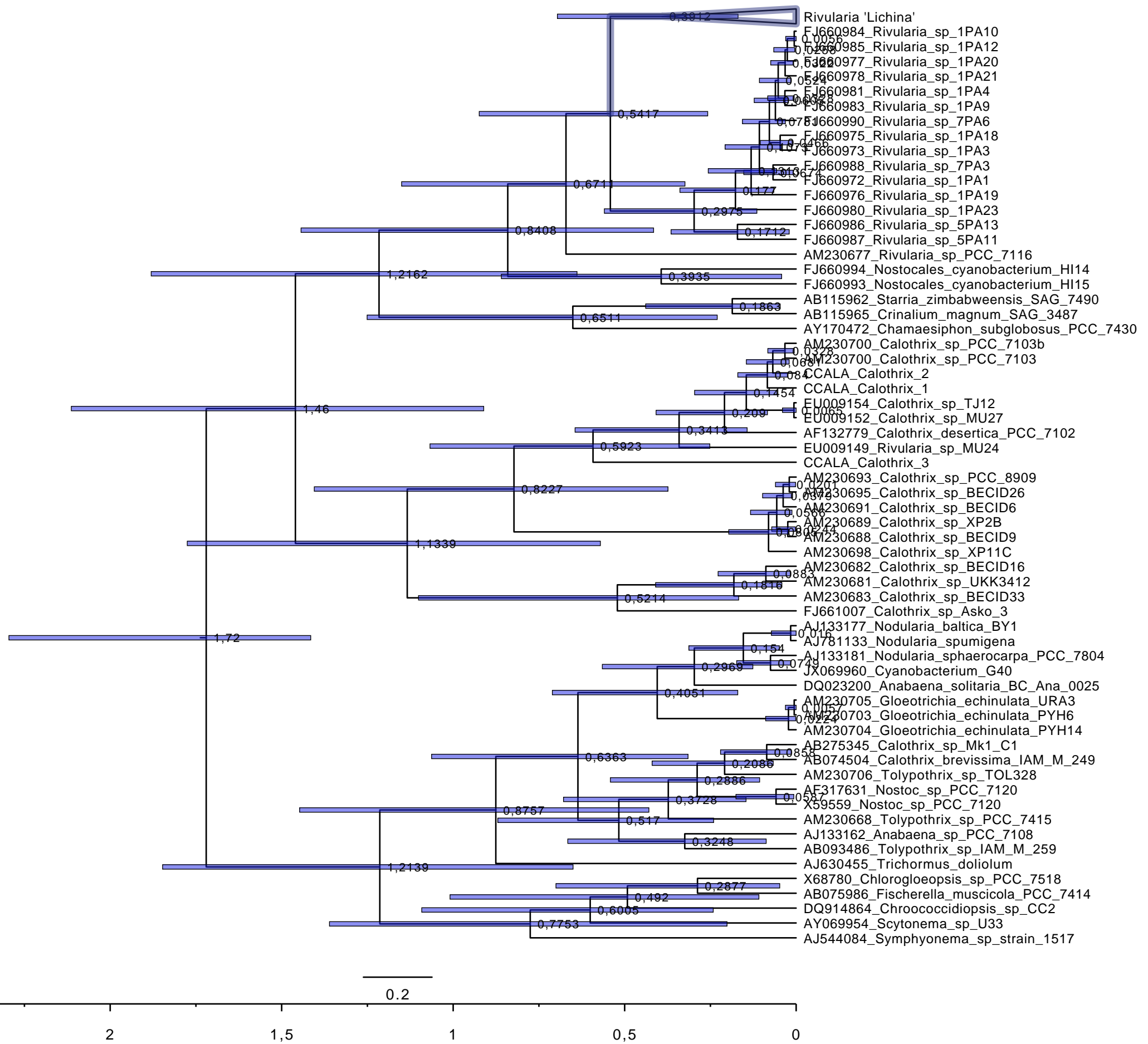

Supplement: S1 Fig — (PDF) [file pone.0132718.s001.pdf]

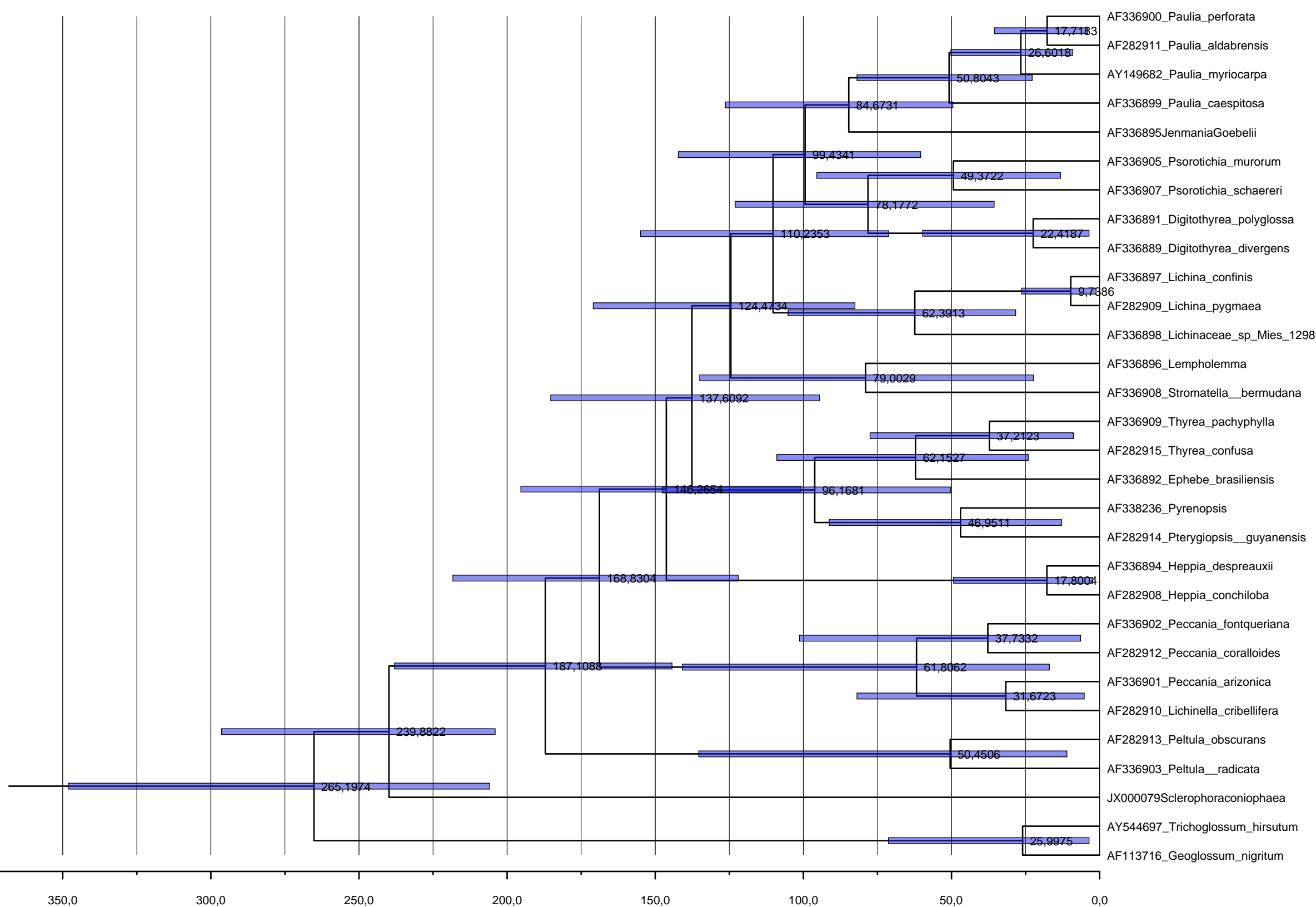

Supplement: S2 Fig — (PDF) [file pone.0132718.s002.pdf]
