# Supplementary material for: Ecological Specialization of Two Photobiont-Specific Maritime Cyanolichen Species of the Genus Lichina
Source: PLoS One. 2015 Jul 16;10(7):e0132718. doi: 10.1371/journal.pone.0132718 (PMC4504470; doi:10.1371/journal.pone.0132718)
Supplement: S1 File — (DOCX) [file pone.0132718.s003.docx]

**Supplementary information S1**: Effect of environmental variables on community structure (Haplotype level).

In *L. confinis* there is a significant difference between CC1 and UK (**), and Gal and UK (*) (same with OTUs). Global R = 0.21 (p<0.01).

In *L. pygmaea* there is a significant difference between CC1 and UK (**), Gal and UK (*). Canary Islands are significantly differentiated (**). Global R = 0.316 (p<0.001).

Using both lichens in the same analysis there are differences between CC1 and UK (**), CC1 and Gal (*), CC1 and ALG (*), Gal and UK (*), Gal and ALG (*), and all the regions and Canary islands (**). Global R= 0.443 (p<0.001).

*Effect of environmental variables on community*

Using both lichens the models obtained are very weak, needing between 8 and 13 variables.

Models for *L. pygmaea* haplotypes use the variables bio7, bio10, chlomin, sstmean, substratum, and latitude. Usually either sstmean or latitude. The variable Chlomin may be equivalent to water clarity.

There are too many posible models for *L. confinis* haplotypes, but there are some with 5 variables (and Rho = 0.517 or Rho= 0.518). Variables are bio10 (mean temperatura of warmest Quarter), chlomin (Chlorophyll A concentration mínimum), cloudmax (máximum nubosity), radiation/parmax/parmean, sstmean (sea surface temperature mean) and substratum.

*Comparison OTUs Haplotypes*

Simmilarity matrices are highly correlated using both lichens (R=0.699 ***), or only *L. pygmaea* (0.742 ***) or *L. confinis* (R=0.742 ***) cyanobiont haplotypes.
