# Supplementary material for: Ecological Specialization of Two Photobiont-Specific Maritime Cyanolichen Species of the Genus Lichina
Source: PLoS One. 2015 Jul 16;10(7):e0132718. doi: 10.1371/journal.pone.0132718 (PMC4504470; doi:10.1371/journal.pone.0132718)
Supplement: S3 Table — (DOCX) [file pone.0132718.s006.docx]

**Supplementary table S3:** environmental variables used in the models generation. Source: WordClim (1-19: Hijmans *et al.* 2005) and Bio-ORACLE (20-45: Tyberghein *et al.* 2012)

| **Number** | **Code** | **Environmental variable** |
| --- | --- | --- |
| 1 | bio_1 | BIO1 = Annual Mean Temperature |
| 2 | bio_2 | BIO2 = Mean Diurnal Range (Mean of monthly (max temp - min temp)) |
| 3 | bio_3 | BIO3 = Isothermality (P2/P7) (* 100) |
| 4 | bio_4 | BIO4 = Temperature Seasonality (standard deviation *100) |
| 5 | bio_5 | BIO5 = Max Temperature of Warmest Month |
| 6 | bio_6 | BIO6 = Min Temperature of Coldest Month |
| 7 | bio_7 | BIO7 = Temperature Annual Range (P5-P6) |
| 8 | bio_8 | BIO8 = Mean Temperature of Wettest Quarter |
| 9 | bio_9 | BIO9 = Mean Temperature of Driest Quarter |
| 10 | bio_10 | BIO10 = Mean Temperature of Warmest Quarter |
| 11 | bio_11 | BIO11 = Mean Temperature of Coldest Quarter |
| 12 | bio_12 | BIO12 = Annual Precipitation |
| 13 | bio_13 | BIO13 = Precipitation of Wettest Month |
| 14 | bio_14 | BIO14 = Precipitation of Driest Month |
| 15 | bio_15 | BIO15 = Precipitation Seasonality (Coefficient of Variation) |
| 16 | bio_16 | BIO16 = Precipitation of Wettest Quarter |
| 17 | bio_17 | BIO17 = Precipitation of Driest Quarter |
| 18 | bio_18 | BIO18 = Precipitation of Warmest Quarter |
| 19 | bio_19 | BIO19 = Precipitation of Coldest Quarter |
| 20 | calcite | Calcite concentration (mol/m³) |
| 21 | chlomax | Chlorophyll A concentration (mg/m³) maximum |
| 22 | chlomean | Chlorophyll A concentration (mg/m³) medium |
| 23 | chlomin | Chlorophyll A concentration (mg/m³) minimum |
| 24 | chlorange | Chlorophyll A concentration (mg/m³) range |
| 25 | cloudmax | Cloud fraction (%) max |
| 26 | cloudmean | Cloud fraction (%) mean |
| 27 | cloudmin | Cloud fraction (%) min |
| 28 | damax | Diffuse attenuation coefficient at 490 nm (m-1) (water clarity) max |
| 29 | damean | Diffuse attenuation coefficient at 490 nm (m-1) (water clarity) medium |
| 30 | damin | Diffuse attenuation coefficient at 490 nm (m-1) (water clarity) minimum |
| 31 | dissox | Dissolved oxygen in seawater(ml/l) |
| 32 | nitrate | Seawater nitrate (μmol/l) |
| 33 | parmax | Photosynthetically Available Radiation (Einstein/m²/day) max |
| 34 | parmean | Photosynthetically Available Radiation (Einstein/m²/day) medium |
| 35 | ph | Seawater pH |
| 36 | phos | Seawater phosphate (μmol/l) |
| 37 | salinity | Seawater salinity (PSS) |
| 38 | silicate | Seawater silicate (μmol/l) |
| 39 | sstmax | Sea Surface Temperature (°C) max |
| 40 | sstmean | Sea Surface Temperature (°C) mean |
| 41 | sstmin | Sea Surface Temperature (°C) minimum |
| 42 | sstrange | Sea Surface Temperature (°C) range |
| 43 | Substratum | Substratum (acidic, basic, volcanic) |
| 44 | Latitude | Latitude in Decimal degrees |
| 45 | Longitude | Longitude in Decimal degrees |
