# Supplementary material for: Ecological Specialization of Two Photobiont-Specific Maritime Cyanolichen Species of the Genus Lichina
Source: PLoS One. 2015 Jul 16;10(7):e0132718. doi: 10.1371/journal.pone.0132718 (PMC4504470; doi:10.1371/journal.pone.0132718)
Supplement: S4 Table — (DOCX) [file pone.0132718.s007.docx]

**Supplementary table S4**. Models generated with correlations linking environmental variables and *Lichina confinis* fotobionts distributions. Variables: 9 = Mean Temperature of Driest Quarter; 15 = Precipitation Seasonality; 23 = Chlorophyll A concentration minimum; 25 = Cloud fraction (%) max; 27 = Cloud fraction (%) min; 30 = water clarity; 31 = Dissolved oxygen in seawater; 33 = Photosynthetically Available Radiation (PAR) max; 34 = PAR medium; 39 = Sea Surface Temperature (max).

| **Models generated** | | | **Environmental variables** | | | | | | | | | |
| --- | --- | --- | --- | --- | --- | --- | --- | --- | --- | --- | --- | --- |
| **% runs** | **Corr.** | **No. Vars** | **9** | **15** | **23** | **25** | **27** | **30** | **31** | **33** | **34** | **39** |
| 59.04 | 0.595 | 5 |  |  |  | x |  | x | x | x |  | x |
| 25.17 | 0.587 | 4 | x | x |  |  |  |  | x | x |  |  |
| 9.80 | 0.589 | 5 |  |  | x | x |  |  | x | x |  | x |
| 3.21 | 0.589 | 5 | x |  |  | x |  |  | x | x |  | x |
| 1.64 | 0.596 | 6 |  | x |  | x |  | x | x | x |  | x |
| 0.80 | 0.588 | 5 |  | x | x |  |  |  | x | x |  | x |
| 0.27 | 0.592 | 5 |  |  |  |  | x | x | x | x |  | x |
| 0.04 | 0.585 | 5 | x |  |  |  | x |  | x | x |  | x |
| 0.01 | 0.596 | 6 |  |  |  | x |  | x | x | x | x | x |
| 0.01 | 0.588 | 5 | x | x |  | x |  |  | x | x |  |  |
|  |  | % models | 40 | 40 | 20 | 60 | 20 | 40 | 100 | 100 | 10 | 80 |
|  |  | % runs | 28.4 | 27.6 | 10.6 | 73.7 | 0.31 | 61 | 100 | 100 | 0.01 | 74.8 |
